# A Rare Case of Concurrent Typhlitis and Clostridioides difficile Colitis in an Acute Myeloid Leukemia (AML) Patient Undergoing Chemotherapy

**DOI:** 10.7759/cureus.104828

**Published:** 2026-03-07

**Authors:** Mhd Baraa Aljouhari, Rand Soudan, Mohamed Feras Ebedin, Hussam Ismael

**Affiliations:** 1 Medicine, Emirates Health Services, Sharjah, ARE; 2 Medicine and Surgery, University of Sharjah, Sharjah, ARE; 3 General Surgery/Thoracic Surgery, American Hospital Dubai, Dubai, ARE; 4 Surgery, University of Sharjah, Sharjah, ARE

**Keywords:** acute myeloid leukemia, clostridioides difficile, neutropenia, neutropenic enterocolitis, typhlitis

## Abstract

Typhlitis (neutropenic enterocolitis) and Clostridioides difficile infection (CDI) are two separate medical conditions. Typhlitis is the inflammation of the cecum and usually occurs in neutropenic patients. CDI is a life-threatening disease of the digestive tract and affects immunocompromised patients. The challenge is when both conditions occur together, especially in patients with hematological malignancies and immune compromise post-chemotherapy. This report of a rare case aims to highlight the co-occurrence of typhlitis and CDI in a patient on chemotherapy after being diagnosed with acute myeloid leukemia (AML). The challenge addressed by this article is how to manage such complex cases in immunocompromised individuals. We report a case of a 41-year-old woman with a history of AML, who presented with fever, abdominal pain, and watery diarrhea after consolidation chemotherapy. Laboratory test results confirmed thrombocytopenia and severe neutropenia. Computed tomography confirmed typhlitis, while stool polymerase chain reaction was positive for toxigenic *C. difficile*. She was successfully treated with intravenous meropenem and metronidazole for typhlitis, alongside oral fidaxomicin for CDI, resulting in a significant improvement in the patient’s condition. This emphasizes the importance of diagnosing and creating a management plan that involves the careful selection of antibiotics that target both conditions without adverse interactions. In conclusion, this case adds to the limited literature on the co-occurrence of typhlitis and CDI, especially in patients with hematological malignancies after chemotherapy. It highlights the need for a high clinical suspicion and an individualized treatment plan for this dual pathology.

## Introduction

Typhlitis, also referred to as neutropenic enterocolitis (NEC), is a life‑threatening inflammatory condition of the cecum and right colon that typically develops in the setting of severe neutropenia due to chemotherapy or within the context of hematological malignancies like acute myeloid leukemia (AML), causing mucosal injury and subsequent polymicrobial invasion of the bowel wall [1-4]. It carries a high mortality rate estimated between 30% and 50% [1]. 

The condition occurs most frequently after intensive chemotherapy for acute leukemia, with regimens containing cytarabine and anthracyclines posing a particularly high risk [5]. The pathophysiology involves a triad of chemotherapy-induced mucosal injury, profound neutropenia, and impaired intestinal motility, which together lead to bacterial translocation and inflammation of the vulnerable cecum [6]. 

A life-threatening differential is Clostridioides difficile infection (CDI), a toxin‑mediated colitis that is strongly associated with prior antibiotic exposure, hospitalization, and older age, presenting from mild diarrhea to fulminant, life‑threatening colitis [4,7,8].

Both diagnoses can present with fever, abdominal pain, and diarrhea in immunocompromised hosts, making them important differentials for one another in patients with neutropenic fever and gastrointestinal symptoms [1,4]. However, their pathogenesis and management strategies differ markedly: typhlitis requires rapid initiation of broad‑spectrum intravenous antibiotics, which is simultaneously a major risk factor for the development of CDI, and nearly all antibiotic classes have been implicated in triggering disease by disrupting the gut microbiota [1,4].

The concurrent presentation of typhlitis and CDI in the same patient is infrequently reported in the literature, with only a few documented cases describing this dual pathology in patients with hematological malignancies [1,4]. This creates a significant gap in evidence-based guidance for clinicians facing this diagnostic and therapeutic dilemma, wherein the broad-spectrum antibiotics required for typhlitis may exacerbate CDI, while inadequate coverage of either condition risks life-threatening complications.

We present this case to highlight the importance of maintaining high clinical suspicion for both entities in neutropenic patients with gastrointestinal symptoms, to illustrate a successful management strategy combining targeted therapy for both conditions, and to contribute to the limited body of literature that can inform clinical decision-making in similar complex scenarios.

## Case presentation

A 41-year-old woman with a known history of AML presented to the hospital in September 2023, complaining of sudden fever and abdominal pain. She had recently completed consolidation chemotherapy. During her assessment, she reported pain localized to the middle and lower abdomen, along with 2-3 days of multiple episodes of watery diarrhea, but denied blood in stool or experiencing nausea or vomiting. On admission, she was started on broad-spectrum antibiotics.

Her abdomen was soft on physical examination and non-distended. On deep palpation, there was tenderness in the lower abdomen with no presence of guarding or rebound tenderness. Initial bloodwork revealed profound neutropenia (WBC 0.4 × 10^9/L, ANC 0). Her platelet count was markedly low at 6 × 10^9/L, consistent with significant thrombocytopenia. The results are summarized in Table 1.

**Table 1:** Initial hematologic parameters demonstrating severe neutropenia and thrombocytopenia

Parameter	Result (×10⁹/L)	Reference Range (×10⁹/L)
WBC	0.4	4.0–11.0
ANC	0	1.5–8.0
Platelets	6	150–400

Stool cultures were negative for common enteric pathogens, including Salmonella, Shigella, and Campylobacter. Multiplex gastrointestinal real-time polymerase chain reaction (PCR) testing using the BioFire FilmArray GI Panel, which targets multiple bacterial, viral, and parasitic pathogens, confirmed the presence of toxigenic Clostridioides difficile (C. difficile). 

**Figure 1:**
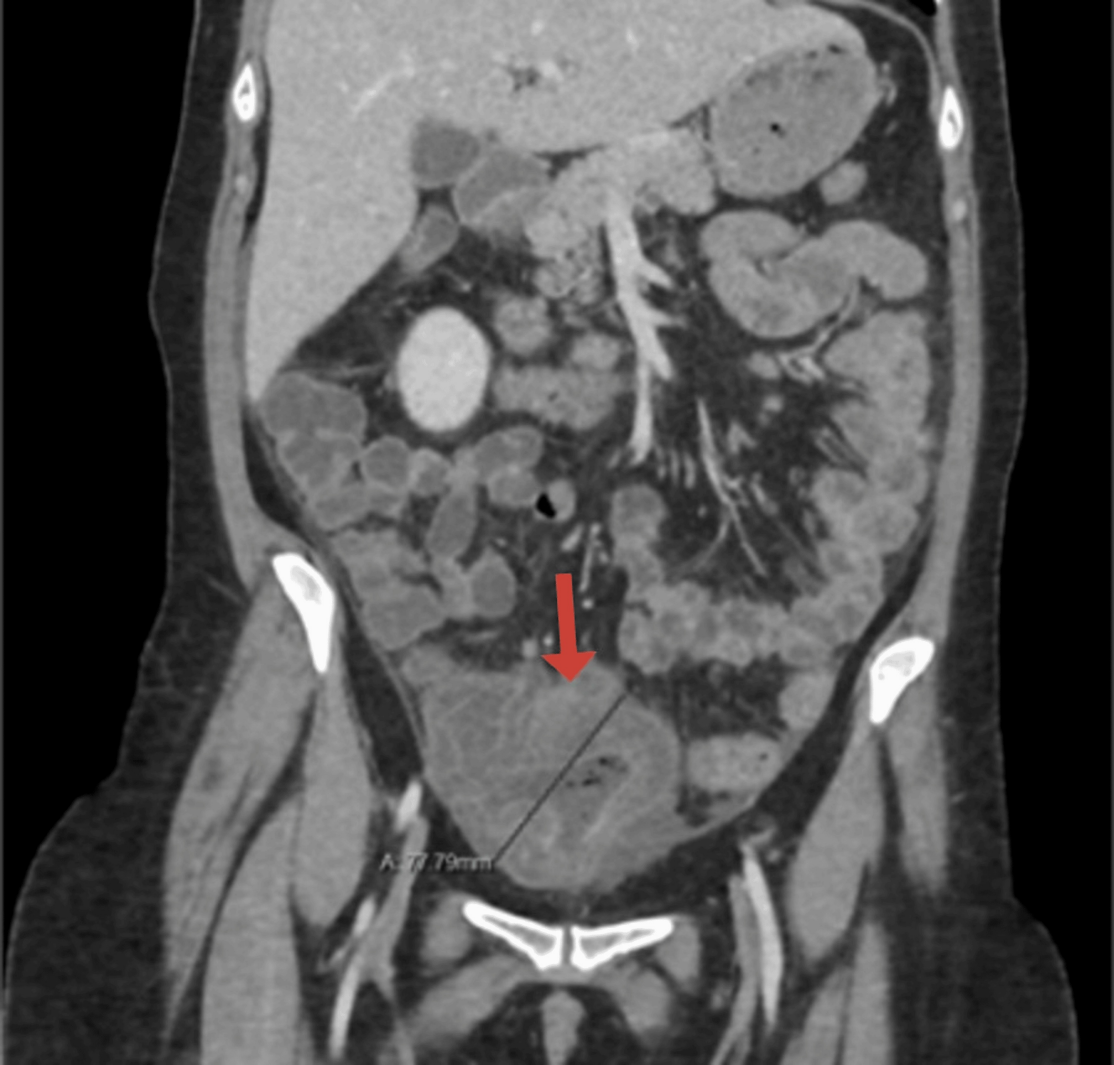
Contrast-enhanced CT of the abdomen (coronal view) Severe thickening and enhancement of the cecal wall with submucosal edema (red arrow), measuring up to 2.2 cm in thickness.

**Figure 2:**
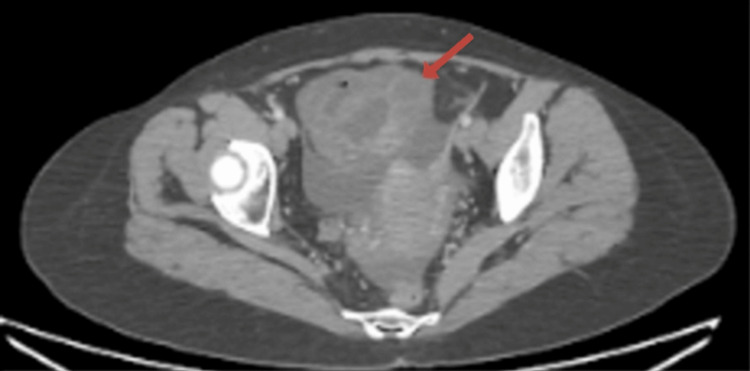
Contrast-enhanced CT of the abdomen (axial view) Circumferential thickening of the cecal wall with mucosal enhancement and submucosal edema (red arrow).

**Figure 3:**
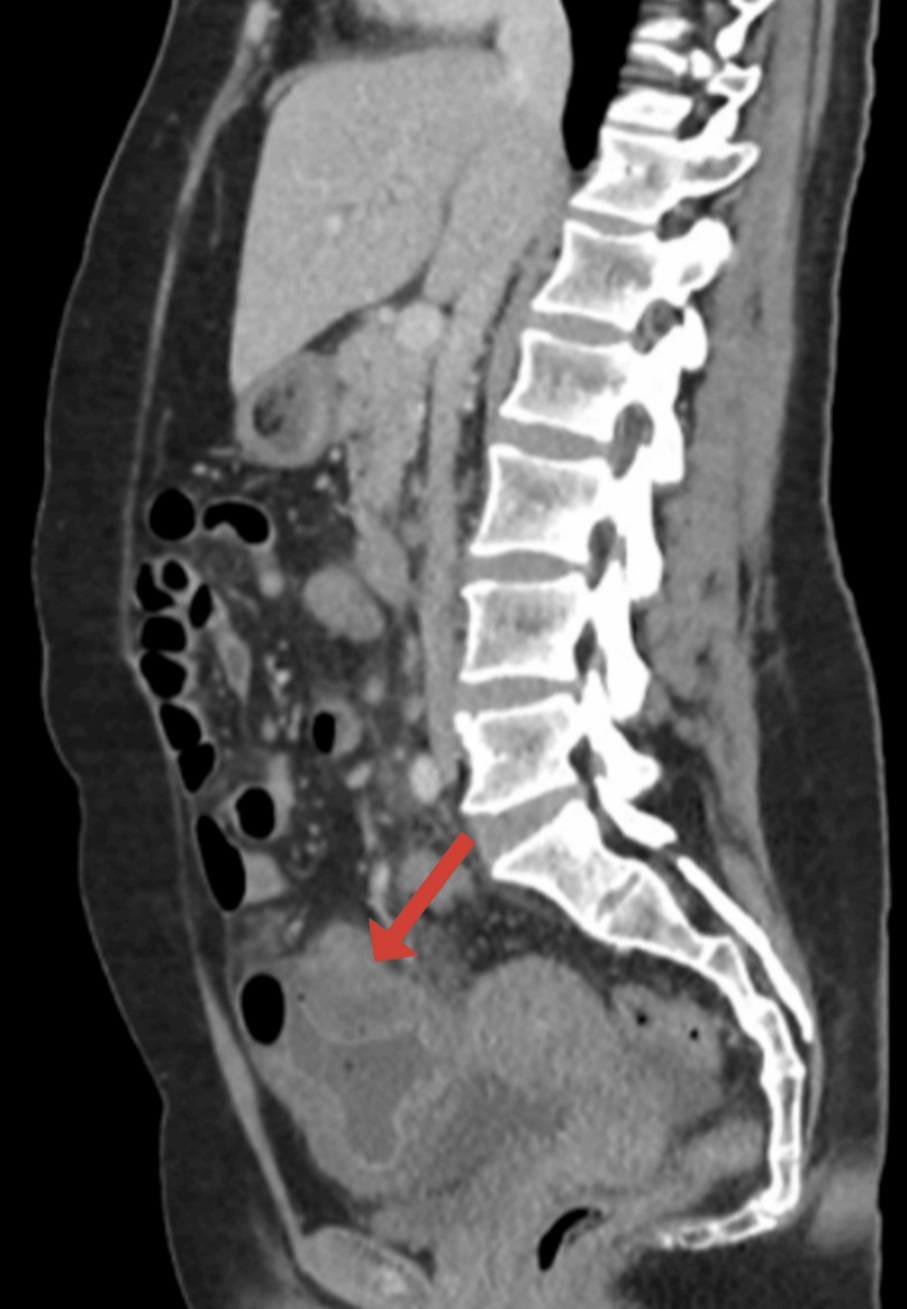
Contrast-enhanced CT of the abdomen (sagittal view) Segmental involvement of the cecum with wall thickening and layered enhancement (red arrow), without evidence of perforation.

A CT abdomen and pelvis with IV contrast (Figures 1-3) demonstrated a distended cecum with thickened walls and surrounding fluid with fat stranding consistent with inflammation. There was no evidence of perforation, as no free air was detected. However, inflammatory changes were seen in other parts of the colon, consistent with NEC.

She was treated for CDI with oral fidaxomicin 200 mg twice daily for 10 days. For NEC, she received intravenous meropenem 1 g every eight hours for 14 days and intravenous metronidazole 500 mg every eight hours for 10 days. Intravenous metronidazole was transitioned to oral administration after five days once the patient demonstrated clinical improvement and tolerance of oral intake. All antibiotics were renally dosed based on normal renal function (creatinine clearance >90 mL/min). The patient was closely monitored, and serial abdominal examinations were performed to assess for clinical signs that would require surgical intervention.

Treatment response was monitored daily using the following parameters: temperature trends, stool frequency, serial abdominal examinations, and complete blood counts.

By the seventh day of hospitalization, the patient showed significant improvement. She had been afebrile for 48 hours, reported fewer than three loose stools daily (down from more than 10 at presentation), and her abdominal examination revealed no tenderness. The platelet count increased to 27 × 10⁹/L, WBC to 2.7 × 10⁹/L, and ANC reached 1.31 × 10⁹/L (Table 2).

**Table 2:** Follow-up hematologic recovery on day seven of hospitalization

Parameter	Result (×10⁹/L)	Reference Range (×10⁹/L)
WBC	2.7	4.0–11.0
ANC	1.31	1.5–8.0
Platelets	27	150–400

Following clinical stabilization, the patient was discharged home on day 10 of hospitalization to complete her 10-day course of oral fidaxomicin. At discharge, she was afebrile, tolerating a regular diet, and having formed stools. She was advised to complete the full course of fidaxomicin and to return immediately for any recurrence of fever, abdominal pain, or diarrhea.

At her one-week follow-up outpatient clinic visit, the patient remained asymptomatic with complete resolution of gastrointestinal symptoms.

## Discussion

Typhlitis, also known as NEC, is a life-threatening condition characterized by inflammation of the cecum, terminal ileum, and right-sided colon. The cecum is most frequently affected due to the combination of intestinal stasis, its high distensibility, and a limited blood supply, which makes it vulnerable to injury. Patients with weakened immune systems, mucosal barrier damage, and neutropenia are at high risk of developing NEC [2,3]. The inflammatory response that occurs in NEC can produce both local and systemic effects [6]. Locally, bowel wall inflammation may lead to perforation, peritonitis, abscess formation, and sepsis [6]. Systemically, the immune dysregulation, characterized by pancytopenia and elevated cytokines, can result in severe bleeding and impaired tissue healing.

Even though there is variability in the incidence reported across studies, NEC cases have increased with the expanded use of intensive chemotherapy [5]. A 2023 review highlighted that regimens containing cytosine arabinoside (cytarabine) and anthracyclines (like idarubicin or daunorubicin), commonly used for AML induction, are associated with a higher incidence of NEC [5]. It is hypothesized that the increased risk is due to the direct injury to the gastrointestinal lining and reduction in gut motility, which predisposes patients to bacterial infections [9]. 

The most common clinical signs of NEC are abdominal pain (especially in the right lower quadrant), diarrhea (which can be bloody and/or watery), nausea, vomiting, and fever [2,3,6]. Careful differential diagnosis is key, given that these symptoms overlap with appendicitis, ischemic colitis, and CDI.

Even though CDI is an important differential, a major challenge is that C. difficile PCR positivity only proves colonization with a toxigenic strain and does not by itself confirm CDI as the sole cause of colitis in a neutropenic patient, so symptoms may be due to NEC, CDI, or dual pathology [7,9,10]. CDI is both a clinical and a laboratory diagnosis, requiring compatible symptoms (typically ≥3 new unformed stools in 24 hours) plus evidence of toxigenic C. difficile using a highly sensitive screening assay (GDH EIA or NAAT/PCR) or free toxins in stool [10,11].

In neutropenic patients with abdominal pain, CDI diagnostics and NEC evaluation must proceed in parallel: CT imaging is the gold standard for NEC, allowing confirmation of bowel wall thickening (>4 mm), mesenteric stranding, bowel dilation, pneumatosis intestinalis, or perforation, with NEC often involving the small bowel, cecum, and ascending colon, whereas CDI usually remains confined to the colon and shows more prominent colonic wall thickening (often >12 mm) [9,10].

In this patient, the involvement of the cecum on imaging, combined with a neutrophil count of 1.3 × 10⁹/L, strongly suggested typhlitis. Additionally, the multiple episodes of loose stools, stool PCR confirming the presence of C. difficile, and CT finding of prominent wall thickening indicate concomitant CDI. Both conditions likely contributed to the patient’s acute abdominal pain and diarrhea, necessitating treatment for both.

Her clinical improvement by day seven coincided with partial hematologic recovery, as shown in Table 2: ANC increased from 0 to 1.31 × 10⁹/L, WBC from 0.4 to 2.7 × 10⁹/L, and platelets from 6 to 27 × 10⁹/L. While antibiotic therapy was essential for controlling both infections, the concurrent rise in neutrophil count likely facilitated definitive clearance of organisms and resolution of mucosal inflammation. This case illustrates that in neutropenic patients, clinical recovery depends on the combination of appropriate antimicrobial therapy and restoration of host immune function. 

The management of NEC in this patient required careful balancing to avoid worsening C. difficile colitis while addressing typhlitis. Although antibiotics are necessary to treat NEC, some antibiotics can exacerbate CDIs. This necessitated a tailored treatment plan to optimize outcomes for both conditions.

Since she did not have signs of peritonitis, bowel perforation, or severe gastrointestinal bleeding, a medical approach for the NEC was pursued. This included bowel rest, hemodynamic support, correction of cytopenia, and administration of broad-spectrum antibiotics targeting Gram-positive organisms, Gram-negative bacteria (including Pseudomonas), and C. difficile [4].

The recommended therapy includes a monotherapy choice with piperacillin-tazobactam or a carbapenem, and an alternative combination option of cefepime or ceftazidime with metronidazole [6]. The patient was given a combination of intravenous metronidazole and intravenous meropenem.

The decision to initiate dual anaerobic therapy was guided by the systematic analysis of NEC in adults [9], which highlights the severity of this condition in immunocompromised hosts and supports the use of broad-spectrum, multidrug regimens in critically ill patients to cover resistant organisms and prevent clinical deterioration [6]. In this context, we elected to provide overlapping anaerobic coverage (meropenem plus metronidazole) until cultures and clinical status clarified the need for continued dual therapy. 

A study on CDIs in neutropenic patients reported a 90.9% response rate to oral metronidazole, making it a reliable first-line option in such cases [12]. However, recent guidelines for patients with hematological malignancies emphasize that for hospitalized or immunosuppressed patients with CDI, initiation of treatment with oral vancomycin or fidaxomicin is often recommended as the preferred agent [13]. Therefore, she was treated for CDI with oral fidaxomicin.

To avoid worsening CDI during NEC treatment, several strategies were employed, including narrowing antimicrobial coverage as soon as culture data permitted, prioritizing CDI-specific therapy with fidaxomicin, which preserves colonic flora, conducting daily reassessment of the continued need for each antibiotic, and closely monitoring diarrhea frequency, abdominal exam findings, and inflammatory markers to detect early signs of CDI flare or treatment failure.

Surgical intervention is considered in severe cases with diffuse peritonitis, pneumoperitoneum, persistent gastrointestinal bleeding despite correction of cytopenia, or failure of medical therapy [14].

To our knowledge, this is one of the few reported cases of concomitant typhlitis and C. difficile colitis in a post-chemotherapy patient with AML. This case highlights the importance of early recognition and heightened clinical suspicion, judicious antimicrobial use, tailored treatment strategies, and a multidisciplinary approach in managing complex, coexisting infections in immunocompromised patients.

## Conclusions

This case demonstrates that concurrent typhlitis and CDI should be considered in neutropenic patients with fever, abdominal pain, and diarrhea following chemotherapy for hematological malignancies. The key diagnostic lesson is the necessity of parallel evaluation with CT imaging for typhlitis and stool PCR with clinical correlation for CDI, as symptoms alone cannot distinguish between these entities. The therapeutic challenge lies in balancing broad-spectrum antibiotic coverage for typhlitis while avoiding exacerbation of CDI, a goal achieved in this patient through combination therapy with meropenem and metronidazole for typhlitis alongside CDI-specific therapy with fidaxomicin. Clinical recovery coincided with hematologic recovery, underscoring that antimicrobial therapy and restoration of host immune function are both essential for optimal outcomes in immunocompromised patients with dual pathology.
